# Targeting the mammalian target of rapamycin (mTOR): a new approach to treating cancer

**DOI:** 10.1038/sj.bjc.6602162

**Published:** 2004-09-14

**Authors:** S Chan

**Affiliations:** 1 Consultant Oncologist, The City Hospital, Nottingham NG5 1PB, UK

**Keywords:** PI3K pathway, PTEN, Akt, m-TOR, CCI-779, Rapamycin

## Abstract

mTOR is a downstream mediator in the PI3K/Akt signalling pathway, which plays a critical role in regulating basic cellular functions. These include cell proliferation, survival, mobility and angiogenesis. Rapamycin and its analogues (CCI-779, RAD001 and AP23573) have specific antagonistic action on the function of mTOR. This leads to inhibition of the downstream signalling elements and results in the cell cycle arrest in the G1 phase. This group of drugs may have a place in Oncology for the treatment of cancers, which occur as a result of increased activity of the PI3 kinase/Akt/m-TOR pathway. The basic structure of the pathway was reviewed in this article, together with results of the clinical studies targeting mTOR for cancer therapy. This is an exciting area for development and poses many challenges to researchers.

## THE PI3-KINASES, AND MTOR

The phosphoinositide 3-kinase (PI3K) family of enzymes is responsible for the production of 3-phosphoinositide lipid second messengers including phosphoinositol trisphosphate (PIP3). The pathway controls a cascade of signals that regulates many basic cellular properties, including proliferation, survival, motility, and angiogenesis. Evidence implicating the role of deregulation of the PI3K in oncogenesis includes the finding that PI3K activity is linked to viral oncogenes such as v-src (Li *et al*, 1997), v-abl, v-ros, ras (Chan *et al*, 1999). Multiple mechanisms for the activation of the PI3K-AKT/PKB pathway have been identified in a range of cancers (Mills *et al*, 2001, 2003). These include mutation and silencing of the pTEN tumour suppressor gene that encodes a lipid phosphatase that reverses the PI3K reaction, and amplification, overexpression and activation of the AKT/PKB. The activated AKT phosphorylates a variety of target proteins. One of these, called BAD, is a protein that normally encourages cells to undergo apoptosis. By phosphorylating BAD, AKT inactivates it, thereby promoting cell survival (Chao and Korsmeyer, 1998). AKT also promotes cell survival by inhibiting other activators of apoptosis, in some cases by inhibiting the transcription of the genes that encode them. Various approaches are being taken to block PI3K signalling in human cancer. One strategy is to inhibit individual oncogenic kinases within the PI3K pathway. The two prototype PI3K inhibitors are wortmannin (a natural product) and the chromenone LY294002, both have shown anticancer activity *in vitro* and in animal models, but have significant limitation for clinical use because of the lack of kinase specificity and metabolic instability (Workman, 2003).

During the mid-1990s, through molecular cloning efforts, a novel family of high molecular mass kinases whose catalytic domains bore a clear resemblance to those of PI3Ks was identified. Based on this primary sequence homology, the newly identified kinases were named PI3K and related kinases (PIKKs). The members of the PIKK family are found in all eukaryotic cells, and accumulating data from genetic, biochemical, and pharmacological studies suggest that these proteins play key roles in fundamental cellular processes, including proliferation and genome surveillance (Hartwell and Kastan, 1994; Paulovich *et al*, 1997). Based on primary sequence alone, it seemed reasonable to predict that the PIKKs were either lipid kinases or dual lipid–protein kinases, but the evidence that PIKKs transmit signals exclusively through the phosphorylation of protein substrate is increasing rapidly. The PIKKs includes three subfamilies: the TOR subfamily, ataxia telangiectasia, the ATM gene product, and the DNA-dependent protein kinase.

The first members of the PIKK family to be molecularly cloned were TOR1 and TOR2 from studies in budding yeast cells (*Saccharomyces cerevisiae*). The isolation of cDNAs encoding a mammalian ortholog, mTOR, followed shortly (Brown *et al*, 1994).

## FUNCTIONS OF THE MAMMALIAN TOR PROTEIN

The members of the Target of Rapamycin (TOR) subfamily are uniquely targeted for inhibition by rapamycin, a feature that has greatly facilitated efforts to understand their functions in eukaryotic cells. Rapamycin is a macrolide antibiotic with antifungal and immunosuppressive properties, which has been approved as an immunosuppressant drug for organ transplantation (Abraham and Wiederrecht, 1996). Rapamycin binds to a highly conserved cytoplasmic receptor FK506-binding protein-12 (FKBP12). This FKBP12-rapamycin complex binds to and inhibits the kinase activities of the TOR protein subfamily, an activity not expressed by either component alone. Rapamycin's mechanism of action represents a solution for the design of a small molecule inhibitor bearing a high level of specificity for a large polypetide target. The FKBP12 receptor positions rapamycin in the optimal orientation to interact with the TOR proteins and also supplies structural determinants that contribute to the affinity and specificity of this interaction.

Studies of rapamycin-treated or TOR-depleted yeast and mammalian cells have shown that loss of TOR function leads to arrest in early G1 phase (Barbet *et al*, 1996). Furthermore, these cells show a severe reduction in protein synthesis resembling those cells in G0 phase, a response normally triggered by starvation. These findings suggested that the TOR proteins play a general role in the translational control of gene expression during cell growth, and the overall rate of protein synthesis in response to nutrient supply and other environmental changes.

Unlike yeast cells, mammalian cells display widely varying sensitivities to the growth-inhibition of rapamycin. A typical target cell for rapamycin is the activated T lymphocyte, which undergoes G1 to S phase progression in response to IL-2 or T-cell growth-promoting cytokines. IL-2-stimulated T cells accumulate in the mid/late G1 phase of the cell cycle in the presence of very low concentration of rapamycin (less than 10 nM) (Morice *et al*, 1993a, 1993b).

## ROLE OF MTOR IN P70 S6 KINASE ACTIVATION AND THE APPLICATION OF THE KINASE AS A BIOLOGICAL END POINT FOR CLINICAL STUDY

There are two proteins whose phosphorylation state has been shown to be regulated by mTOR. The first targeted protein is p70 s6 kinase, a serine-threonine kinase that is activated in response to a broad range of mitogenic stimuli. Rapamycin blocks both the phosphorylation and activation of p70 s6K in mammalian cells. The second end point for the mTOR-dependent phosphorylation pathway is the translational-repressor protein PHAS-1 (also termed 4E-BP). Both proteins participate in the regulation of protein synthesis in cells stimulated by either mitogens or hormones.

The regulation of p70 s6k by upstream protein kinases is complex (Proud, 1996). It is clear that treatment with rapamycin quickly and efficiently inhibits the *de novo* phosphorylation of p70 s6k induced by hormonal stimuli, as well as the phosphorylation of previously activated p70 s6k. The level of p70 s6k activity in peripheral blood mononuclear cells has been showed to correlate with the efficacy of RAD001, an mTOR inhibitor in clinical studies (Lane, 2003). It has been suggested that this biomarker strategy can be used to define the minimal effective dose for clinical studies of mTOR inhibitors

**Figure 1:**
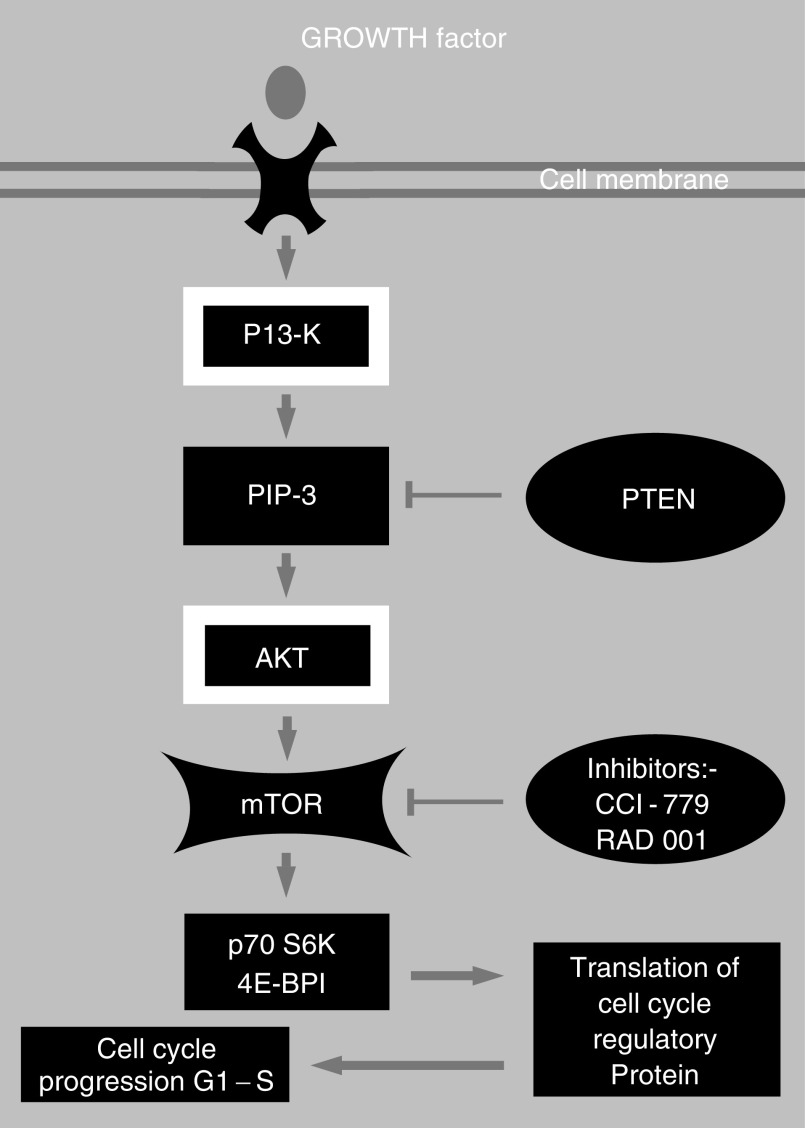
A diagram of the mTOR pathway.

In summary, both PI3K and its downstream serine-threonine kinase AKT participate in mTOR activation by growth factors such as hormones and insulin (Gingras *et al*, 1998; Scott *et al*, 1998). This places mTOR in a signalling cascade implicated in the abnormal growth and survival of certain cancer cells. Therefore, rapamycin or other mTOR kinase inhibitors may have significant anticancer activity in tumours driven by activated PI3K or AKT or both (Dancey, 2000) (Figure 1

).

## MTOR INHIBITORS IN CLINICAL DEVELOPMENT

Results of clinical studies had been reported using the three rapamycin analogues: RAD001 (Novartis) (Lane, 2003), AP23573 (ARIAD Pharmaceuticals) (Mita *et al*, 2004), and CCI-779 (Wyeth) (Yu *et al*, 2001). The pharmacological action of these rapamycin analogues, like rapamycin, is mediated through its binding to the intracellular protein FKBP12 and subsequent inhibition of the protein kinase mTOR.

Sirolimus, the major metabolite of CCI-779, also binds to FKBP12. Exposure to the sirolimus metabolite is substantial, with a mean value of approximately 1.4- to 2.3-fold that of CCI-779 following i.v. administration to as high as 13.8- to 34.5-fold the values seen following oral administration. It is, at present, unclear what proportion of the anticancer activity of CCI-779 is due to CCI-779, or its metabolite sirolimus. Clinical development on the use of mTOR inhibitors in oncology is advancing rapidly. CCI-779 was the first to enter the field. Results from its clinical studies may contribute to understanding some of the challenges involved in designing trials for this group of drugs.

### CCI-779: preclinical studies

A study of a panel of breast cancer cell lines showed differences in the sensitivity to the antiproliferative effects of CCI-779 (Yu *et al*, 2001). Sensitive cell lines were oestrogen receptor positive, or lacking in expression of the tumour suppressor gene pTEN and/or overexpression of the oncogene Her-2/neu. CCI-779 inhibited mTOR functions in both sensitive and resistant cell lines. Treatment of sensitive lines with CCI-779 resulted in a decrease in cyclin-D and c-myc levels and an increase in p27 Kip-1 levels. There was a correlation between the activation of the Akt pathway and sensitivity to CCI-779. These results suggest that mTOR may be a good target for the treatment of breast cancer. Especially in patients with tumours that are driven by the activation of the Akt pathway. The overexpression of the Akt pathway maybe due to an amplification of Her-2/neu oncogene, or oestrogen receptor dependence, or the loss of the pTEN tumour suppressor function.

### CCI-779: clinical studies

The proposed mechanism of action by CCI-779 as an inhibitor of mTOR, and its properties as a cytostatic agent may be useful in clinical oncology. A Phase 1 study where the drug was given once a week intravenously (Alexandre *et al*, 1999) antitumour activity was demonstrated in patients with advanced breast cancer in the study.

## BREAST CANCER

A phase 2 safety and efficacy trial of two levels of CCI-779 in women with advanced breast cancer was initiated in 2001. Enrolment was completed in February 2002 with 109 patients recruited (Chan *et al*, 2003a). The majority of patients received more than two lines of prior chemotherapy, including anthracyclines and/or taxanes. Results of the study were presented at the American Society of Clinical Oncology annual meeting in 2003. There was no significant difference in terms of efficacy between the patients who received 75 or 250 mg week^−1^ doses. However, toxicity was higher in the 250 mg week^−1^ group and a dose reduction was required in 45.1% of patients in this group compared with 28.6% in the 75 mg week^−1^ group. The most common reason for study discontinuation was lethargy and depression (75 mg, *N*=5/42; 250 mg, *N*=12/43). Adverse events were generally grade 1 or grade 2 and often resolved without discontinuing treatment. Grade 3 and 4 toxicities were rare, and included: depression, mucositis, diarrhoea, transiently raised GGT, hyperglycaemia, hypokalaemia, leucopenia, and infection. In terms of efficacy, among the 98 evaluable patients, objective response rate (WHO criteria) was 10% (10/98), and the median response duration was 5.4 months (95% CI: 3.8–7.2). Clinical benefit (partial response and stable disease over 2 months) was noted in 36 patients (37%).

The most striking finding from the first analysis of the biological phenotype (as reported by individual centres) of those breast cancers in the study, was that none of the 32 Her-2 negative tumours showed any significant response to the treatment with CCI-779. The value of this study is the demonstration of significant clinical activity of CCI-779, with manageable toxicities, in advanced breast cancer.

We studied tumours preserved in paraffin blocks from our 28 patients who participated in the trial using immunohistochemical methods (Sharma *et al*, 2003). Our results showed that 4/28 tumours were negative for PTEN (indicative of gene mutation), and 3/4 of these tumours showed objective response to treatment with CCI-779; 3/28 tumours had HER-2 gene overexpression (3+ Herceptin Test or FISH +ve), 2/3 of these tumours showed an objective response to the drug. These findings suggest that PTEN mutation and/or HER-2 overexpression in breast cancer may predict response to mTOR inhibitors.

Using immunohistochemical staining of tissue microarrays, we have also studied the expression of PTEN (wild type) and its relationship with phosphorylated AKT, as well as with other recognized prognostic factors (HER-2 expression, oestrogen receptor status, histological grade, lymph node status at initial surgery, tumour size, age, menopausal status, risk of recurrence, and survival) in 429 cases of operable invasive breast cancer with a minimum 5 years of follow-up (Chan *et al*, 2003b). We found that 7.5% of cases did not express wild-type PTEN, while 70 and 22.5% of cases were weakly and strongly positive, respectively. The immunohistochemical expression of PTEN was related to pAKT levels (*P*<0.001) – tumours with low levels of pAKT frequently did not express wild-type PTEN. No significant correlation was observed between PTEN and other known prognostic factors or patient survival. pAKT expression was inversely related to tumour grade (*P*<0.001) and size (*P*<0.002), while a positive correlation was seen with ER status (*P*<0.001). pAkt expression was not however significantly related to patient survival nor was it an independent prognostic factor. These data suggest that the relationship between pAKT and PTEN is complicated in invasive breast cancer, and that better understanding of the pathway is necessary before we can define the predictive markers for response to treatment with the mTOR inhibitor.

The role of PI3K/AKT/mTOR pathway may play an important role in the development of Tamoxifen resistance in breast cancer. Activation of the oestrogen receptor can drive the PI3K pathway (Simoncini *et al*, 2000). Cross talk between erb-1 and ER has been shown to activate the pathway, which has been associated with oestrogen-independent transcriptional activity (Aronica and Katzellenbogen, 1991; Pietras *et al*, 1995; Smith, 1998; Campbell *et al*, 2001), and breast cancer cell lines with activated Akt is resistant to the growth inhibitory effects of Tamoxifen (Degrafenried *et al*, 2002). The combination of antioestrogens and mTOR inhibitors may improve the therapeutic index of either agent alone. A phase 2 study comparing the use of Letrozole with or without CCI-779 in patients with advanced breast cancer has recently completed recruitment (Baselga *et al*, 2004), but it is too early to assess the efficacy of this combination.

## RENAL CELL CARCINOMA

In a randomised double-blind phase 2 study (Atkin *et al*, 2002); weekly CCI-779 at three different doses (25/75/100 mg) was given once per week intravenously to patients with previously treated advanced renal cell carcinoma. Toxicity profile was similar to that reported from the breast cancer trial. Side effects were largely grade 1 and 2. Overall response rate was 5%. Interestingly, there was no significant difference between the three dose levels in terms of efficacy, but the 25 mg group has the least toxicity reported.

The combination of CCI-779 and interferon alpha was studied in patients with advanced renal cell carcinoma (Dutcher *et al*, 2003). The result showed that the combination was well tolerated, and partial response to the treatment was observed. A large phase 3 study comparing the combination *vs* interferon alpha alone in this patient population has already been started.

## OTHER CLINICAL STUDIES USING MTOR INHIBITORS: IN OTHER TUMOUR TYPES AND USED IN COMBINATIONS WITH CYTOTOXIC CHEMOTHERAPY

Preliminary efficacy data from the phase 1 single-agent studies indicate antitumour activities (in addition to breast cancer and renal cell carcinoma) of mTOR inhibitors (CCI-779, RAD001 and AP23573) in patients with non-small-cell lung cancer, anaplastic astrocytoma, mesothelioma, soft tissue sarcoma, cervical and uterine cancer (O'Donnell *et al*, 2003; Mita *et al*, 2004).

There is evidence to suggest that mTOR inhibitors may be used to overcome drug resistance to cytotoxic chemotherapeutic agents. For example, treatment of Doxorubicin-resistant and PTEN defective prostate cancer cells with CCI-779 has been shown to reverse Doxorubicin resistance (Grunwald *et al*, 2002). There is no clinical data available on this combination, at the present. CCI-779 combinations with Gemcitabine and 5-Fluorouracil (+Leucovorin) were limited by gastrointestinal events (diarrhoea, mucositis, and nausea). In combination with Gemcitabine (day 1 and 8 of 21days cycle), the maximum tolerated dose of CCI-779 was only 15 mg per sq m, and the weekly i.v. dose in the 5-Fluorourcil combination was 75 mg per sq m (Wyeth, personal communication).

## SUMMARY

The mTOR inhibitors have demonstrated clinical activities in a broad spectrum of solid tumours. Unresolved challenges to researchers include: definition of the optimal dose and scheduling; identifying the appropriate biological end points for dose finding studies; finding predictive markers for tumour phenotypes likely to response to mTOR inhibitors; and finding optimal combination treatment with other drugs and radiotherapy. Drug development targeting the mTOR pathway looks exciting, but a lot more work remains to be done.
